# Influence of light-exposure during late incubation and early feeding on the performance of layer chicks in a multitasking test and step detour test

**DOI:** 10.1016/j.psj.2025.106203

**Published:** 2025-12-05

**Authors:** Catharina M.H. Broekmeulen, Sabine G. Gebhardt-Henrich, Yamenah Gómez, Michael J. Toscano

**Affiliations:** a Centre for Proper Housing: Poultry and Rabbits (ZTHZ), Division of Animal Welfare, Vetsuisse Faculty, University of Bern, Burgerweg 22, CH-3052 Zollikofen, Switzerland; b Graduate school of Cellular and Biomedical Sciences, University of Bern, Mittelstrasse 43, CH-3012 Bern, Switzerland; c Centre for Proper Housing: Poultry and Rabbits (ZTHZ), Federal Food Safety and Veterinary Office, Burgerweg 22, CH-3052 Zollikofen, Switzerland

**Keywords:** Laying hen chick, Laterality, On-farm hatching, Dual task, Detouring, Footedness

## Abstract

During on-farm hatching, chicken embryos experience continuous light during late incubation, and have immediate access to feed and water post-hatch. While on-farm hatching compared to hatchery-hatching improves several welfare indicators in broilers, the combined effects of light exposure and early feeding on behavioral and cognitive development in broilers and layers remains unclear.

On-farm hatching systems are expected to expand into the laying hen sector due to the advances regarding *in ovo*-sexing and vaccination technologies. We investigated effects of continuous light exposure from 18 until 21 days of incubation and early feeding on multitasking abilities and visuomotor lateralized behavior in layer chicks. Using a two-by-two factorial design, 1,280 hatching eggs were assigned to one of four different groups of factor combinations with early feed and water access (FW) and continuous light exposure during late-stage incubation (L): FW+/L+, FW-/L+, FW+/L- or FW-/L-. In total, 128 focal chicks were subjected to behavioral testing. A multitasking test assessed vigilance towards an overhead predator while foraging at 10 to 13 days of age. L+ chicks identified the predator faster than those incubated in darkness (*p* < 0.0001) and resumed pecking faster after detection (*p* = 0.006), suggesting better multitasking abilities in both FW+/L+ and FW-/L+ chicks. These findings suggest enhanced behavioral flexibility and resilience in L+ chicks, which may improve adaptation to daily stressors and overall welfare during rearing. A step detour test assessed footedness when crossing a step and detour direction (i.e., laterality proxies) at five and seven weeks of age. L+ chicks exhibited weaker footedness compared to L- chicks. FW+ chicks showed more right-side foot and left-side detouring preferences, indicating greater left-hemispheric engagement and a more optimistic response to testing. Detour preferences also shifted with age, possibly reflecting motivation to approach the social stimulus. Differences in side-preferences may result from light-induced modulation of lateralization during late incubation, influenced by interactions with other asymmetries or perinatal environmental factors. However, the mechanisms underlying these interactions remain unclear and warrant further investigation.

## Introduction

Under natural brooding conditions, variation in embryonic light exposure during incubation occurs whenever the hen leaves the nest to eat and drink or to turn the eggs (Rogers, 1995; Archer and Mench, 2014). When the hen leaves the nest during the last three days of incubation, external light can only stimulate the right eye due to the embryo’s position in the egg (Koshiba et al., 2003). Consequently, light stimulation of the right eye and thereby the left hemisphere of the brain induces lateralization of the visual pathways (i.e., thalamofugal visual pathways to the Wulst region) and associated behavior (Rogers, 1995; Deng and Rogers, 2002). Additionally, visual lateralization improves aspects of cognition (Vallortigara et al., 2011), such as focused attention, visual discrimination (Andrew and Rogers, 1991; Rogers, 2010), and specific abilities, like food-searching and predator detection in a dual task (Rogers et al., 2004; Dharmaretnam and Rogers, 2005; Wichman et al., 2009). It has also been hypothesized that lateralization increases left hemispheric control of behavior, which makes it more likely for chicks to express positive cognitive biases in response to stressful stimuli (Rogers, 2010). Several studies, most of which were performed in broiler chicks, support this hypothesis by showing that visually lateralized chicks habituate quicker, show milder responses to stressors (Chiandetti et al., 2013), and are less fearful compared to non-lateralized chicks (e.g., Sui et al., 1997; Andrew et al., 2009; Rogers, 2012; Archer and Mench, 2017). Whereas chicks that lack visual laterality struggle to inhibit one hemisphere over the other, their consequent multitasking abilities may be affected, making them more prone to show negative cognitive biases towards stimuli (Daisley et al., 2009; Rogers, 2010). Combined with limited ability of dark-incubated chicks to process both spatial and object specific cues (Chiandetti et al., 2005), this could explain why dark-incubated chicks perform poorly on dual tasks involving foraging and raptor detection, and show strong distress responses (Rogers et al., 2004; Dharmaretnam and Rogers, 2005; Wichman et al., 2009). Taken together, these studies support that lighted incubation in commercial hatcheries can be used as a strategy to improve welfare by generating visual laterality and an enhanced control of behavioral response by the left hemisphere (Rogers, 2023).

In on-farm hatching systems, hatching eggs (i.e., chicken embryos) are transported to rearing barns on day 18 of incubation. The chicken embryos are exposed to continuous light from placement in the barn on day 18 until day 21 of incubation (De Jong et al., 2019, 2020; Molenaar et al., 2023), and after hatching, chicks have immediate access to feed and water (van de Ven et al., 2009; Giersberg et al., 2021; Souza da Silva et al., 2021). The combination of hatching and brooding phases in on-farm hatching systems may more closely resemble natural early life conditions, where chicks explore and forage near the nest (Bateson, 1966, Hemsworth and Edwards, 2021, Hogan, 1973, Rogers, 1995). Immediate access to feed and water post-hatch provides chicks with the opportunity to forage, which has been hypothesized to act as environmental enrichment, stimulating cognitive development and influencing emotional lateralization or reactivity to stressful events (Hollemans et al., 2018; Campbell and Lee, 2021). However, there are knowledge gaps regarding how the hatching system factors – independently and interactively - affect the behavioral and cognitive development of layer chicks during on-farm hatching. Our study aim was to investigate how behavioral development, with a focus on multitasking abilities and visuomotor lateralized behavior in laying hen chicks was affected in the period up to eight weeks of age. We assessed two treatment factors: 1) continuous light exposure during late incubation (L), and 2) immediate feed and water access post-hatch (FW), and their interactions in a 2×2-factoral design, which was replicated twice. The resulting factor combinations were exposures to: FW+/L+, FW-/L+, FW+/L-, or FW-/L- (fully deprived). Our design allowed us to identify the importance of the two treatment factors in their contribution to the behavioral development of layer chicks. A multitasking test assessed the ability to perform two tasks simultaneously (i.e., foraging: pebble vs. grains, and vigilance for aerial predators), and eye preferences to spot and sustain view of a predator. A retention test assessed if chicks had learned and retained the ability to differentiate between pebbles and grains during the foraging task. A step detour test assessed foot preferences when crossing a step, and detour preferences when circling around a barrier, as well as fearfulness, based on latency to leave the start box and time needed to finish the test. It was hypothesized that FW+/L+ and FW-/L+ chicks would show different behavioral responses and side preferences compared to FW+/L- and FW-/L- chicks. Furthermore, we wanted to explore whether immediate feed and water access (i.e., FW) had modulating effects on side preferences and behavioral responses.

## Material and methods

### Experimental design and housing

The study was performed in an experimental barn at the Aviforum in Zollikofen, Bern, CH, and consisted of two replicated trials, with major procedural details described elsewhere (Broekmeulen et al., 2024; 2025). The first trial ran from June until August 2022, and the second trial ran from October until December 2022. A total of 1280 hatching eggs were acquired from Prodavi SA (Switzerland) to be hatched on-farm. The eggs for each trial came from Super Nick hybrid-parental flocks (H&N International, Germany) with a reproductive age of 32 to 33 weeks. The animals were housed in an experimental barn without natural daylight to minimize pen variation in light exposure and the behavioral tests were performed in an adjacent room. The treatment exposure period lasted from day 18 until day 21 of incubation and started as soon as the eggs were haphazardly assigned to one of the factor combinations (Table 1).

**Table 1:** Two-by-two factorial design with factors: feed and water access (FW) and light exposure (L). The treatment exposure period lasted from day 18 until day 21 of incubation.

**Factors and levels**	**Feed and water access (FW)**	
**Light exposure (L)**	**+**	**-**
+	**+ / +**	**+ / -**
-	**+ / -**	**- / -**

The pens were arranged into two rows where one row had eight pens and the other nine. Sixteen out of the seventeen pens were pens (four pens / factor combination group) with the remaining one available as a sick pen if needed. In all pens, the walls were covered with visual barriers (height: 155 cm) and were equipped with a ramp (116×24 cm, angle: 40°) between the raised tier (plastic grid, 102×103×72.5 cm) and litter area (108×103 cm). Pens also contained perches (one at tier level, one raised 5.5 cm above the tier), lights (22 W, 3000 K, 2400 lm, SILOX, Once Animal Lighting, Signify, The Netherlands), and drinker lines. A single round feeder provided *ad libitum* feed. During the first week, the first tier was covered with chick paper and litter was provided on top of the tier. The chicks were given access to the litter area after they were seven days of age.

During the treatment exposure period (i.e., the last three days of incubation, embryonic days 18 to 21), the barn was separated into a dark and light section by tarpaulin that hung from the ceiling between the two rows. The tarpaulin was removed after the treatment exposure period. Multiple light intensity measurements were taken with a lux-meter (MAVOLUX 5032C/B USB, GOSSEN GmbH, Nürnberg, Germany) at egg level (e.g., in the hatching tray) both before the hatching eggs arrived and during the treatment exposure period to confirm that no light reached the eggs. In the dark section, the readings gave <0.01 lux. In the light section, the readings gave 50 to 60 lux depending on the angle.

### On-farm hatching and chick processing

The hatching eggs were kept under complete darkness if light exposure was not included in the treatment. Upon arrival, groups of 40 eggs were assigned to each pen. The eggs were placed in the hatchery trays (67×57 cm; HatchTech B.V., Veenendaal, NL) on bricks to raise the trays 20 cm above the tier. The barn environmental system was programmed to maintain an air temperature of 32°C, relative humidity of ≥ 30 %, and air flow < 0.15 m/s. The barn climate was monitored from the time at which eggs were set until 8:00 h in the morning on day 21 of incubation. In addition, the on-farm hatching was monitored by measuring the eggshell temperatures of three marked eggs per pen with an infrared thermometer (MEDISANA TM 750, MEDISANA GmbH, Neuss, Germany), where the objective was to maintain egg temperatures between 36°C and 38°C. The climate settings were adjusted whenever the eggshell temperatures deviated from the interval.

The hatching window was closed at 8:00 h in the morning on day 21 after which the chicks were taken from the pens and processed according to commercial procedures by hatchery professionals. The processing procedures consisted of counting, sorting, feather sexing, grading, and vaccinating. After processing, the chicks were placed into pen-specific crates to prevent mixing of groups. The female chicks stayed in the barn and were vaccinated according to a commercial program. After all processing steps were completed (approximately four hours), the selected female chicks were relocated to a new home pen to ensure that experimenters were blinded during behavioral testing and to minimize room side confounds. The goal was to obtain approximately 80 female chicks per factor combination, with a maximum of 20 chicks per pen to simulate Swiss commercial rearing densities. However, the total number of chicks per factor combination varied due to differences in hatching rates (Table 2).

**Table 2:** Number of female chicks per factor combination group for each replicate trial.

	**Number of female chicks**		
**Factor combination group**	**Trial 1**	**Trial 2**	**Total**
**FW+/L+**	70	64	134
**FW-/L+**	78	63	141
**FW+/L-**	70	70	140
**FW-/L-**	71	71	142

The surplus of chicks was transported back to the hatchery to be sold or killed according to commercial standards (i.e., by CO_2_ exposure) resulting in only female chicks used for behavioral tests. After the treatment period ended, all chicks were managed according to the same flock rearing management plan.

Although, our focus was on the hatching conditions of female layers, we hatched both male and female chicks which required chick sexing. Our study was designed according to the 3R principles. We considered the male to female ratios as well as mortality during incubation. Table 3 gives an overview of the hatchability per factor combination group per trial. The male to female ratio was 53.0 to 47.0 % for trial 1, and 45.5 to 54.5 % for trial 2.

**Table 3:** Hatchability for each factor combination group.

**Factor combination group**	**Hatchability (%)**
**FW+/L+**	96.3
**FW-/L+**	95.1
**FW+/L-**	95.3
**FW-/L-**	95.3

Male chicks were sampled for stress hormones (see Broekmeulen et al., 2024), this resulted in an overall reduced number of hatching eggs that needed to be incubated, and thus surplus one-day old male chicks that had to be transported back to the hatchery to be killed (i.e., methods are described in Broekmeulen et al., 2024).

### Behavioral testing

At one day of age, after allocation to the final home pen, four female chicks per pen (*N* = 64 focal chicks/trial) were selected as focal animals for behavioral testing. In addition, four companion chicks were selected from the same home pen. Each focal and non-focal companion chick duo was tagged with flexible numbered leg rings (Flexiringe, Fieger AG, Untertuttwil, Switzerland) for identification. The leg rings were replaced by gradually larger leg rings with the same number at seven, 14, and 35 days of age.

### Multitasking and retention test

The multitasking test compared the chicks’ ability to attend to two tasks simultaneously – foraging for grains on a pebbled background while simultaneously being vigilant for aerial predators. The retention test assessed the chicks' abilities to retain the ability to discriminate between grains and pebbles during the foraging task, and to determine whether exposure to a predator had affected their learning of the task.

**Fig. 1:**
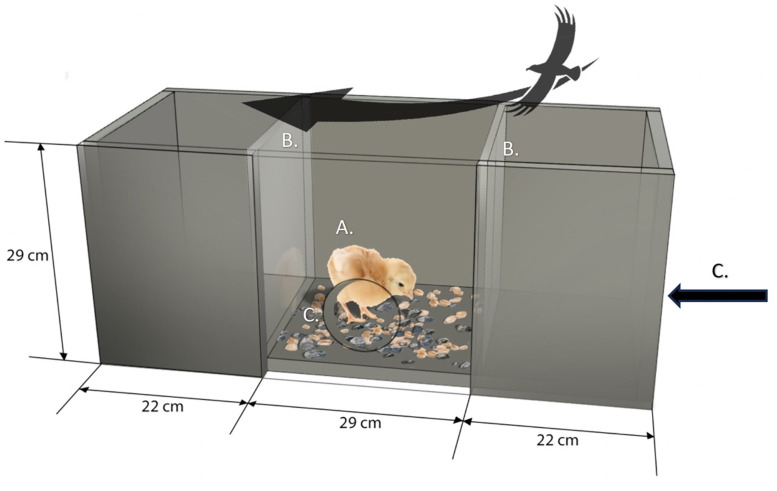
Multitasking Test Arena with dimensions. The chicks were subjected to a dual task in the central compartment of the arena. There was a pebbled floor in the central compartment with chick mash scattered over top. A. Wooden panel with an opening at the floor level of the arena for live observations, B. Plexiglas panels. C. Camera positions for behavioral observations.

Habituation to the multitasking test arena (Fig. 1) started when the chicks were three days of age and continued until 10 days of age. Two test arenas were used simultaneously during habituation for reasons of expediency. From three to five days of age, two chick duos from the same pen were placed in the arenas at the same time. From five days of age onwards, only one chick duo was placed in the arena at the time. The duos were placed in the central compartment of the arena for a five-minute period. The floor tile in the central compartment of the arena was covered with chick paper. As soon as the five minutes were over, the duos were rewarded with a grape piece, and the chicks were removed from the arena after it was consumed. Tests started after 10:00 h in the morning at 10, 11, 12, and 13 days of age. For expediency reasons, we only tested four (one pen / group) out of the sixteen pens on each of the testing days. The chicks were fasted for three hours before the tests started by staggered removal of the feeders. Only one arena was used during testing, and the chick paper was removed from the floor tile exposing the pebbled floor. Chick mash was scattered over the pebbled floor in a 3:5 of mash to pebbles by volume. The tiles were weighed before and after the test to assess how many grams of chick mash were consumed by the chick duos. Chick droppings were removed before weighing after the test. Each chick duo was placed in the central compartment of the arena. The test started as soon as the chicks’ feet touched the floor tile. The duos were observed live on camera, and as soon as the focal chick in the duo performed five pecks, predator silhouettes (wooden, wingspan: 11 cm) were presented overhead with a zipline at an interval of 18 seconds as a simulation of a soaring bird of prey. Video recordings were made and later used to observe eye preferences and behavioral responses of the focal chicks. The measured responses were eye preference to first spot the predator and eye preference to sustain view of the predator, feed intake in grams for each duo during the test, foraging behavior, fear/vigilance behavior, and comfort/displacement behaviors (Table 4). The test was stopped when a total of 20 predator silhouettes had been presented.

**Table 4:** Ethogram during the multitasking and retention test.

Behavioral class	Behavior	Description
Foraging	Pecking	Using beak to tap on object to search for food
	Scratching	Backwards raking motion with one of two feet
Fear/ Vigilance	Vocalization	Startle call, peeping
	Interruption of pecking	Ceasing to peck combined with looking up at the predator
	Crouching	Freezing while squatting / flattening body to the ground
	Flight	Running / turning away/ jump
	Defecation	Fecal matter excreted
Comfort / Displacement	Preening	Tidying or cleaning feathers with beak

The same chick duos were subjected to the retention test at 14 and 15 days of age. The catching, transportation and test procedures were similar as described above in the Multitasking test section. The same multitasking test arena was used during the retention test, although no predators were presented during the retention test. The measured responses were the total number of pecks at the chick mash, time needed to perform 30 pecks and feed intake in grams during the test. The test was stopped after the focal chick performed a total of 30 pecks or after five minutes. The chicks passed the retention test criterion when they performed 30 pecks at grains within five minutes

### Step detour test

The step detour test was developed as a multidimensional test to assess lateralized behavior: foot preferences to cross a step and detour preferences when circling around a barrier (i.e., proxies for brain lateralization), with major procedural details described elsewhere (Broekmeulen et al., 2025). Habituation to the step detour test arena started when the chicks were 22 days of age and continued until 26 days of age. Before habituation started, a wooden board was placed in the test arena in front of the barrier as a barricade. One out of four focal chicks per pen was placed in the start box at the time, after which the guillotine door was opened which gave the chick access to the arena.

During the first day of habituation, the latency to leave the start box was measured. The focal chicks were subjected to the step detour test at 29 days of age. For testing, four focal chicks and two non-focal chicks from the same pen were taken to the experimental room. The non-focal chicks were used as a social stimulus during the test and placed in a box opposite to the start box before the test started. The non-focal chicks had access to feed within their box during the test. One of the four focal chicks at a time was taken out of the crate to be placed in the start box. After 60 seconds the guillotine door was opened giving the chick access to the whole arena.

During the test, the chicks were required to leave the start box, cross a step (height: 10 cm; width: 40 cm), detour around a barrier (i.e., mesh) and step into a drawn rectangle behind the barrier to complete the test. The measured responses during the step detour test were latency to leave the start box, foot preference while crossing over the step, detour direction, and time to reach the rectangle. The focal chicks were subjected to the step detour test in two trials: every day consecutively during the fifth week of age (i.e., 29 to 34 days of age) and again during the seventh week of age (i.e., 43 to 48 days of age).

### Laterality index

For the analysis of the step detour test, we first calculated laterality indices for two trials: at five and at seven weeks of age to assess foot preferences and detour preferences. The laterality index (LI) was calculated with following formula (Whiteside et al., 2018):

LI=(left−−right)/(left+right)

In this formula, left or right represents the count of which foot a chick chose to cross the step or direction the chick chose to detour around the barrier. The LI values are proportions that reflect the direction of preference, as well as the strength or degree of preference. With only six observations per individual per trial, the only possible LI values were: -1, -0.7, -0.3, 0, 0.3, 0.7 and 1. Positive LI values reflect a left side preference, while negative LI values reflect a right-side preference. LI values of 0 reflect that the chicks did not show a preference. The strength of laterality was classified into three categories (i.e., absolute proportions): based on the absolute LI values of 1, 0.7 or 0.3, which reflected strong preference, intermediate preference, or weak preference, respectively.

### Statistical analysis

The statistical analyses were carried out with R (R Core Team, Version 4.4.0 (2024-04-24)) using RStudio (Rstudio Team, Version 2024.04.2+764). The limit for statistical significance was set to P < 0.05. The response variables for latency to spot the predator, and latency to return to pecking were classified as numeric responses and analyzed using negative binomial models due to overdispersion (Bates et al., 2015). Eye preference to spot the predator, eye preference to sustain view of the predator, and meeting retention test criterion were classified as binary responses and analyzed using a binomial linear mixed-effects model. The response variables for feed intake in grams during the multitasking test and retention test were classified as numeric and analyzed using a mixed Poisson regression model. For the above-mentioned response variables, factor feed and water and factor light exposure were included into the two-way interaction models as fixed factors with two levels each (i.e.: FW+, FW-; L+, L-), and chick ID was nested in pen, nested in trials as a random term. The factor combinations were applied at pen level. Therefore, pen was regarded as the experimental unit, while chick served as the observational unit. However, for feed intake specifically, chick duo represented the observational unit. Changes in eye preference between spotting the predator and sustaining view of the predator, and behavioral observations are presented as descriptive data in the results section.

The step detour test data were analyzed in three stages. Firstly, we assessed if the chicks showed a preference (i.e., LI ≠ 0). Foot preference and detour preference were classified as binary responses and analyzed using binomial linear mixed-effects models. Secondly, we created subsets of data with all chicks that showed foot and of all chicks that showed detour preferences.

Both strength (degree) of foot preference and detour preference were assessed using absolute LI values. Strength of foot preferences were collapsed into two categories: weak foot preferences (equal to 0.3) or intermediate to strong foot preferences (above 0.3), because strong foot preferences were underrepresented in the dataset – only 6 % of chicks showing a foot preference (11 out of 179) fell into this category. Strength of detour preferences were divided into two categories: weak to intermediate (below 1) or strong (equal to 1), because weak preferences were underrepresented in the dataset (16 % of chicks; 39 out of 232). The strengths of preferences were classified as binary responses and analyzed using binomial linear mixed-effects models. Lastly, the same subsets were analyzed to assess whether chicks showed a left or right-directional foot preference and detour preference. The directional preferences were categorized as binary responses and analyzed using binomial linear mixed-effects models. All step detour test models included main factors: FW, L, and week of age (two levels: 5, 7), as well as their two-way interactions and three-way interactions as fixed effects. Moreover, chick ID was nested in pen, nested in trial as a random term. Here again, pens were regarded as the experimental unit. The LIs for each individual focal chick were plotted and visually examined to determine whether chicks showed a foot preference, detour preference, and right- or left side preference for both weeks, at five and seven weeks of age.

Latency to leave the start box on the first day of habituation was classified as a numeric response and analyzed using a generalized linear mixed effects model. Latency to leave the start box was only measured once, therefore, only treatment was included as a fixed effect, and pen was nested in trial as a random term. Duration to finish was classified as a numeric response and analyzed using a negative binomial linear mixed effects model due to overdispersion. The chicks got considerably faster over time which caused a lack of variance in the data after 30 days of age. Therefore, the duration to finish was only compared between 29 and 30 days of age.

The model included main factors: FW, L, and day of age (two levels: 29, 20), as well as their two-way interactions and three-way interactions as fixed effects. Moreover, chick ID was nested in pen, was nested in trial as a random term.

The full models were reduced to the final model by stepwise backwards model reduction using likelihood ratio tests (LRT) for model comparisons (ANOVA; lme4 package). To check model assumptions the normality distribution of residuals and homoscedasticity for linear mixed effects models and uniformity and dispersion for Poisson and binomial mixed effects models were visually inspected with Q-Q plots and/or tested with the package DHARMa (Hartig, 2018). Subsequently, Tukey’s *post hoc* tests were performed (Holthorn et al., 2008) to determine differences between levels of the fixed factors. When the residuals were non-random, the raw data were transformed and re-evaluated. Finally, the odds ratios and 95 % CI were backtransformed where needed for presentation of results. Interactions and main effects are described in the results section only if they were statistically significant, and test statistics of non-significant factors are listed in Tables 6 and 7.

## Results

### Multitasking and retention test

**Fig. 2:**
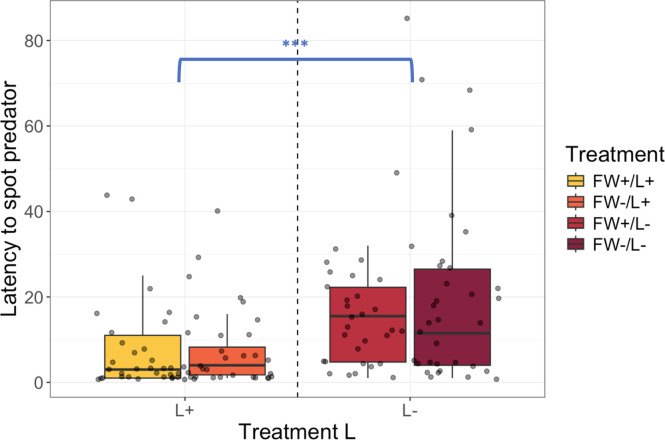
Latency to spot predator. L+ chicks were 41.2 % faster to spot the predator compared to L- chicks across both FW treatment levels (ratio = 0.412, 95 % CI [0.28,0.62], *p* < 0.0001). FW = immediate post-hatch access to feed and water; L = continuous light exposure from day 18 until day 21 of incubation ***p < 0.0001.

Latency to spot the predator silhouette was affected by L (*X^2^* = 15.12, df =1, *p* = 0.0001) whereas FW did not account for additional variance (*X^2^* = 0.0002, df =1, *p* = 0.99). Latency to spot the predator was not affected by an interaction between L and FW (*X^2^* = 0.05, df =1, *p* = 0.83). L+ chicks were 41.2 % faster to spot the predator compared to L- chicks across both FW treatment factor levels (ratio = 0.412, 95 % CI [0.28,0.62], *p* < 0.0001) (Fig. 2).

**Fig. 3:**
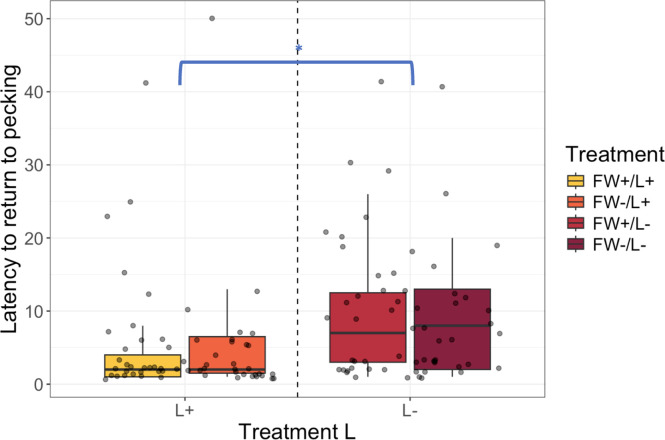
Latency to spot predator. L+ chicks were 52.8 % faster to return to pecking after first spotting the predator compared to L- chicks independent of FW factor levels (ratio = 0.528, 95 % CI [0.35, 0.81], *p* = 0.0031). FW = immediate post-hatch access to feed and water; L = continuous light exposure from day 18 until day 21 of incubation *p = 0.01.

Latency to return to pecking after first spotting the predator silhouette was affected by L (*X^2^* = 7.58, df = 1, *p* = 0.006), whereas FW did not account for additional variance (*X^2^* = 2.61, df = 1, *p* = 0.11). Latency to spot the predator was not affected by an interaction between L and FW (*X^2^* = 2.06, df =1, *p* = 0.15). L+ chicks were 52.8 % faster to return to pecking compared to L- chicks across both FW levels (ratio = 0.53, 95 % CI [0.35, 0.81], *p* = 0.003) (Fig. 3).

During the multitasking test, all 128 chicks used alarm calls after spotting the predator. Regardless of factor combinations, 26 chicks crouched, 11 chicks defecated, 32 chicks showed escape attempts, three chicks dustbathed, and 27 chicks preened.

Descriptive results for eye preferences to spot the predator, eye preferences to sustain view of the predator, feed intake per duo during the multitasking test, and chicks that met the test criterium and feed intake per duo during the retention test are summarized in Table 5. The test statistics for these parameters were not influenced by factors FW, L, or interactions between the two and are summarized in Table 6.

**Table 5:** Descriptive results for eye preferences to spot the predator, eye preferences to sustain view of the predator, feed intake per duo (i.e., focal and companion chicks) during the multitasking test, and chicks that met the test criterium and feed intake per duo during the retention test for each treatment factor level.

**Parameter**	**Treatment factor levels**			
	**Light exposure during incubation**		**Feed and water access**	
	**L+**	**L-**	**FW+**	**FW-**
**Multitasking test**				
**Eye preference to spot predator**	Left: 64.1 % (*n* = 41) Right: 35.9 % (*n* = 23)	Left: 51.6 % (*n* = 33) Right: 48.4 % (*n* = 31)	Left: 53.1 % (*n* = 34) Right: 46.9 % (*n* = 30)	Left: 62.5 % (*n* = 40) Right: 37.5 % (*n* = 24)
**Eye preference to sustain view of predator**	Left: 50.8 % (*n* = 31) Right: 49.2 % (*n* = 30)	Left: 44.6 % (*n* = 25) Right: 55.4 % (*n* = 31)	Left: 52.5 % (*n* = 32) Right: 47.5 % (*n* = 29)	Left: 42.9 % (*n* = 24) Right: 57.1 % (*n* = 32)
**Average feed intake for duos (g)**	64.4 (SE = 54.2)	80.0 (SE = 58.2)	63.8 (SE = 43.8)	78.6 (SE = 65.4)
**Retention test**				
**Chicks that met the test criterium**	Yes: 82.8 % (*n* = 53) No: 17.2 % (*n* = 11)	Yes: 84.4 % (*n* = 54) No: 15.6 % (*n* = 10)	Yes: 82.8 % (*n* = 53) No: 17.2 % (*n* = 11)	Yes: 84.4 % (*n* = 54) No: 15.6 % (*n* = 10)
**Average feed intake for duos (g)**	43.0 (SE = 35.8)	32.8 (SE = 22.7)	37.3 (SE = 36.0)	38.4 (SE = 23.5)

**Table 6:** Test statistics for eye preferences, feed intake, and chicks that met the test criterium during the multitasking and retention test that were not significantly influenced by FW, L, or their interactions. FW = immediate feed and water access post-hatch; L = continuous light exposure from 18 until 21 days of embryonic development.

Parameter	Factor	Statistical Output
Multitasking test		
Eye preference to spot the predator	FW * L	*X*^2^ = 1.79, df = 1, *p* = 0.18
	FW	*X*^2^ = 1.06, df = 1, *p* = 0.32
	L	*X*^2^ = 1.78, df = 1, *p* = 0.18
		
Eye preference to sustain view of the predator	FW * L	*X*^2^ = 1.65, df = 1, *p* = 0.20
	FW	*X*^2^ = 0.97, df = 1, *p* = 0.32
	L	*X*^2^ = 0.47, df = 1, *p* = 0.49
Feed intake	FW * L	*X*^2^ = 0.09, df = 1, *p* = 0.77
	FW	*X*^2^ = 0.72, df = 1, *p* = 0.40
	L	*X*^2^ = 2.22, df = 1, *p* = 0.14
Retention test		
Number of chicks that met the criterion	FW * L	*X*^2^ = 0.02, df = 1, *p* = 0.89
	FW	*X*^2^ = 0.03, df = 1, *p* = 0.87
	L	*X*^2^ = 0.03, df = 1, *p* = 0.87
Feed intake	FW * L	*X*^2^ = 0.02, df = 1, *p* = 0.89
	FW	*X*^2^ = 0.51, df = 1, *p* = 0.47
	L	*X*^2^ = 2.78, df = 1, *p* = 0.10

### Step detour test

#### Strength and direction of foot preference

Whether chicks showed a foot preference was affected by interactions between L and FW as well as between L and week of age (*X^2^* = 3.88, df = 2, *p* = 0.05; *X^2^* = 4.31, df =1, *p* = 0.04, respectively). Across factor combinations, 62.5 % of FW+/L+ chicks (*n* = 40), 76.6 % of FW-/L+ chicks (*n* = 49), 75.0 % of FW+/L- chicks (*n* = 48), and 65.6 % of FW-/L- chicks (*n* = 42) showed a foot preference to cross the step. In L- chicks, foot preferences increased from five to seven weeks of age, with an increase from 56.3 % to 75.0 % in FW-/L- chicks (*n* = 18 to *n* = 24), and an increase from 68.8 % to 81.3 % in FW+/L- chicks (*n* = 22 to *n* = 26). In L+ chicks, foot preferences were stable in FW-/L+ chicks with 75.0 % (*n* = 24) showing a foot preference at five weeks of age and 78.1 % (*n* = 25) showing a foot preference at seven weeks of age, whereas the number of foot preferences decreased in FW+/L+ chicks, with 71.9 % (*n* = 23) at five weeks of age to 53.1 % (*n* = 17) at seven weeks of age. However, the *post-hoc* comparisons did not reveal significant differences the L and FW factor combinations, nor across L and weeks of age interactions.

Strength of foot preference was affected by L (*X^2^* = 5.99, df = 1, *p* = 0.01), but not by FW or weeks of age (*X^2^* = 0.83, df = 1, *p* = 0.36; *X^2^* = 0.11, df = 1, *p* = 0.74, respectively). L+ chicks were 57.0 % more likely to show a weak foot preference instead of an intermediate or strong foot preference compared to L- chicks independent of FW (OR = 0.43, 95 % CI [0.22,0.84], *p* = 0.01).

Direction of foot preference was affected by FW (*X^2^* = 4.90, df =1, *p* = 0.03) but not by L (*X^2^* = 0.89, df =1, *p* = 0.35). FW+ chicks were 49.0 % more likely to have a right-side foot preference to cross the step compared to FW- chicks independent of L level (OR = 0.51, 95 % CI [ 0.28,0.93], *p* = 0.03). Additionally, direction of foot preference tended to be affected by week of age (*X^2^* = 3.69, df = 1, *p* = 0.06), with chicks being 45.0 % more likely to have a right-side foot preference at seven weeks of age compared to five weeks of age (OR = 0.55, 95 % CI [0.30, 1.02], *p* = 0.06).

#### Strength and direction of detour preference

Whether chicks showed detour preferences was affected by weeks of age (*X^2^* = 12.34, df =1, *p* < 0.001). At seven weeks of age, 124 focal chicks showed a detour preference (96.9 %), whereas at five weeks of age, 108 focal chicks showed a detour preference (84.4 %), (OR = 6.01, 95 % CI [1.91, 18.9], *p* = 0.002). Treatment factors FW and L did not affect chicks’ detour preferences (*X^2^* = 127.48, df =1, *p* = 0.25; *X^2^* = 1, *p* = 0.17, respectively).

Strength of detour preference was affected by weeks of age (*X^2^* = 15.96, df = 1, *p* < 0.0001), with chicks being three times more likely to show a stronger detour preference at seven weeks of age compared to five weeks of age (OR = 2.94, 95 % CI [1.72, 5.04], *p* = 0.0001). Strength of detour preference was not affected by FW or L levels (*X^2^* = 0.03, df = 1, *p* = 0.87; *X^2^* = 0.31, df =1, *p* = 0.58, respectively).

Direction of detour preference was affected by FW (*X^2^* = 4.26, df =1, *p* = 0.04), with FW+ chicks being 2.55 times more likely to have a left-side detour preference to circle around the barrier compared to FW- chicks independent of L levels (OR = 2.55, 95 % CI [1.09, 5.98], *p* = 0.03). Direction of detour preference was not affected by L or weeks of age (*X^2^* = 1.20, df =1, p =0.27; *X^2^* = 0.68, df =1, *p* = 0.41).

#### Latency to leave the start box and duration to finish the step detour test

Latency to leave the start box on the first day of habituation was not affected by treatment factors FW, L, or their interactions, see Table 7.

**Table 7:** Test statistics for latency to leave the start box on the first day of habituation of the step detour test that were not influenced by FW, L, or their interactions. FW = immediate feed and water access post-hatch; L = continuous light exposure from 18 until 21 days of embryonic development.

**Parameter**	**Factor**	**Statistical Output**
Latency to leave the start box on the first day of habituation	FW * L	*X^2^* = 0.03, df = 1, *p* = 0.86
	FW	*X^2^* = 0.03, df = 1, *p* = 0.87
	L	*X^2^* = 0.19, df = 1, *p* = 0.66

Latency to leave the start box during testing was affected by an interaction between the two treatment factors FW and L, and day of age (*X^2^* = 8.29, df = 1, *p* = 0.004). Duration to finish the test was affected by day of age (X^2^ = 83.09, df = 1, *p* < 0.001) and showed a tendency for a FW influence (*X*^2^ = 3.42, df = 1, *p* = 0.06). *Post-hoc* tests of means for latency to leave the start box during testing and duration to finish the test are summarized in Table 8.

**Table 8:** Summary of multiple comparisons of means for latency to leave the start box during testing and duration to finish the test during the step detour test.

Factor combination	Percentage	Odds ratio	95 % CI	p-value
Latency to leave the start box at 29 days of age *vs.* at 30 days of age				
FW+/L+	65 % shorter	0.35	[0.17, 0.72]	< 0.001
FW-/L+	77 % shorter	0.23	[0.30,1.22]	0.37
FW+/L-	55 % shorter	0.45	[0.22, 0.91]	0.01
FW-/L-	80 % shorter	0.20	[0.09, 0.43]	< 0.001
Duration to finish the test at 29 days of age *vs.* at 30 days of age				
FW+/L+	54 % shorter	0.46	[0.23, 0.91]	0.01
FW-/L+	70 % shorter	0.30	[0.15, 0.58]	<0.001
FW+/L-	62 % shorter	0.38	[0.19, 0.75]	<0.001
FW-/L-	72 % shorter	0.28	[0.14, 0.56]	<0.001

## Discussion

### Multitasking and retention test

On an individual level, visually guided lateralization is thought to be beneficial as it enhances cognition by making individuals more efficient to perform more than one task at the same time (Vallortigara and Rogers, 2005). For this experiment, we adopted a dual task paradigm, a multitasking test, which was designed to engage the left hemisphere in foraging and the right hemispheres in predator detection and recognition (Vallortigara and Rogers, 2005). Based on other studies that used a comparable test (Rogers, 2000; Rogers et al., 2004; Dharmaretnam and Rogers, 2005; Wichman et al., 2009), we hypothesized that light-incubated chicks would be more capable of performing two tasks simultaneously, while dark-incubated chicks would struggle to find grain and detect the predator at once. Therefore, it was expected that light-incubated chicks would have shorter latencies to spot the predator and return to pecking compared to dark-incubated chicks. Additionally, we wanted to investigate whether immediate post-hatch feed and water access modulated emotional lateralization resulting from light-induced left-hemispheric dominance.

In support of our hypothesis, we observed that light-incubated chicks were faster to interrupt pecking and turn their heads upwards to look at the predator compared to dark-incubated chicks.

The shorter latencies indicate that light-incubation affects vigilance since both FW+/L+, and FW-/L+ chicks were faster to detect the predator compared to dark-incubated chicks. Most chicks stopped pecking as soon as they first detected the predator, which was often followed by startle calls. However, L+ chicks were found to return to pecking earlier than L- chicks, which suggests that FW+/L+ and FW-/L+ chicks were less distracted by the predator when foraging. Taken together, the better ability to forage for grains while being vigilant for predators suggests that the chicks from both light-incubated groups had an enhanced ‘efficiency’ in cognitive tasks that require multitasking (Rogers et al., 2004). The shorter latencies to return to pecking after spotting the predator might also indicate that L+ chicks have an increased ability to control negative behavioral responses and/or assess if a novel stimulus in the environment could pose a threat ​(Daisley et al., 2009; Rogers, 2010). For instance, the shorter latencies in chicks from both light-incubated groups might indicate that they are more flexible as they modified their behavior in response to the predator during the test. Furthermore, the shorter latency to return to pecking in FW+/L+ and FW-/L+ might reflect dynamic interactions between stimulus-driven processing and instruction-driven processing (i.e., guided by learned instructions) (Manns and Ströckens, 2014; Rogers, 2014)​. In this context, stimulus-driven processing refers to hemispheric specializations for handling specific types of stimuli, whereas instruction-driven processing involves shifts in attention or arousal between the hemispheres, which redirects hemispheric engagement in responding to those stimuli.

The foraging involved in immediate post-hatch feed and water access most likely provides cognitive enrichment to chicks, which might modulate negative behavioral responses to a predator model (Hollemans et al., 2018; Campbell and Lee, 2021). However, based on our findings, we cannot conclude whether immediate post-hatch feed and water access has influenced emotional lateralization, as the FW+ chicks did not show differences in behavior during the multitasking test. Therefore, the potential effects of early feed and water access on emotional lateralized behavior merits further investigation.

In a commercial setting, higher behavioral flexibility could aid chicks to better adapt to stressful aspects of their daily environment and thereby improve welfare during the rearing period.

The chicks did not show clear differences in eye preferences to assess the predator when first detecting it, nor did they show eye preferences to sustain their view of the predator after this initial detection, which corresponds with reports by ​Dharmaretnam and Rogers (2005)​. The absence of observed eye preference in the chicks might be attributed to the binocular testing conditions. These binocular conditions allowed the chicks to use either eye to detect the predator, potentially switching between both eyes to evaluate whether the stimulus posed a threat. The observed flexibility might have masked the favor of one eye over the other. The possibility to switch between eyes when foraging might also explain why we did not find differences in feed intake between treatment factors combinations. However, it is still surprising that the shorter latency to return to pecking did not result in higher feed intake of FW+/L+ chicks. Moreover, it should be considered that both focal and companion chicks foraged during the test. Testing multiple animals simultaneously may lead to competition which could distort cognitive performance ​(Mendl, 1999). Additionally, social learning, facilitated by the presence of a companion, might explain why all chicks were able to learn to differentiate between pebbles and grains and perform similarly across factor combinations during the retention test, and meet the test criterion. Social foraging is more natural in chicks than foraging by a single chick which would not occur outside experimental conditions.

Our findings support the hypothesis that lighted incubation contributes to enhanced multitasking abilities in chicks, since the latency responses in light-incubated chicks with and without immediate feed and water post-hatch were similar. At the same time, it is still unclear if and how early feeding might have contributed or interacted with lighted incubation because our findings did not confirm additive effects of early feeding on multitasking abilities in FW+/L+ or FW+/L- chicks. However, caution should be taken when generalizing the results as the feed and water treatment did not involve extreme feed deprivation after hatch, which is more commonly observed under commercial conditions due to the hatchery processing and transportation of one-day old chicks. We previously found that feed deprivation in layer chicks during on-farm hatching leads to similar body weights after 48 hours (Broekmeulen et al., 2024).

### Step detour test

Light exposure during late incubation is known to modulate visual lateralization and visuomotor behavior in chicken ​(Manns, 2022)​. The step detour test involved multiple dimensions of brain lateralization. Therefore, we anticipated differences in foot and detour preferences (i.e., FW+/L+ and FW-/L+ vs. FW+/L- and FW-/L- factor combinations).

Our findings partially support the hypothesis, with L affecting the strength of foot preferences and FW influencing their direction. However, these findings should be interpreted with caution, as we did not observe clear differences in the presence or absence of foot preference across factor combinations. This may reflect limited variability in the foot preferences rather than limited replication, as our study included eight pens per factor combination and the models accounted for trial effects.

### Strength of foot preference

We found that L+ chicks were more likely to show a weak foot preference whereas L- chicks were more likely to show a medium or strong foot preference. However, it is unclear what the functional benefit of a certain strength footedness is during the stepping task, as foot preferences could be context or task dependent.

In multiple parrot species, there is evidence that strength of lateralization is related to better test performance in food discrimination and string-pulling tests (for an overview: Rogers, 2017). However, the link between foot usage when crossing an obstacle and lateralization might be less evident, as it does not involve foraging or manipulation of an object. Whiteside et al. (2018) assessed foot preferences in pheasants with a stepping test and linked strong foot preferences while stepping up to a lower survival rate, which suggests that strong foot preferences, resulting from extreme lateralization, are undesirable in a natural environment (Whiteside et al., 2018). Since chickens are a *Galliformes*-species like pheasants, strong footedness might also be a disadvantage or constraint in chickens. However, a higher strength of laterality in one task might be beneficial to the detriment of another. Therefore, a better understanding of the costs and benefits of lateralization across different contexts is needed.

Interestingly, Tommasi and Vallortigara (1999) suggested that chicks may show a foot preference primarily for postural control/stability rather than manipulative activities like ground scratching, which have a preferred foot for postural control rather than manipulative activities. The differences in laterality relating to activity emphasizes the importance of the foot that remains on the ground over the one used for motor activities (Tommasi and Vallortigara, 1999). However, this difference probably does not apply to the stepping task, since it does not involve a manipulative element. Therefore, we assume that the stepping tasks require both stability and mobility (for an overview: Gabbard and Hart, 1996). However, it remains unclear whether stability or mobility is more demanding for chicks when crossing a step, especially considering that the step height in this study was only 10 cm. Therefore, we recommend selecting behavioral tests that involve performing isolated and manipulative tasks, when assessing footedness in chicken in future studies.

Strength of lateralization and side preferences could potentially be influenced or modulated by other sensory modalities (Ströckens et al., 2013). For instance, asymmetrical vestibular inputs have been shown to affect motor laterality in mice, which led to the hypothesis that an early imbalance in the inner ear could potentially contribute to the development in handedness in humans (Antoine et al., 2018). A similar phenomenon could be observed in chicks, since chicken embryos are exposed to mechanical vibrations during egg processing and transportation on day 18 of incubation (Nordquist et al., 2022), which probably stimulates the vestibular system. In addition, chicken embryos might receive proprioceptive and tactile stimulation from 18 days of embryonic development onwards, partly due to self-stimulation when moving in the egg (Rogers, 1995). Rogers (2023) suggested that visual laterality resulting from light exposure during incubation might interact with other sensory modalities such as those of an auditory and/or olfactory nature. Light-induced lateralization could interact with other asymmetries that develop and/or are enhanced or suppressed by other environmental factors during the perinatal period. For instance, the embryos might have secreted corticosterone in response to environmental stressors during hatching egg transportation (e.g., mechanical vibrations, noises), which might modulate the induction of visuomotor laterality (Rogers and Deng, 2005; Rogers, 2006, Nordquist et al., 2022). One could argue that the current study technically began before day 18 of incubation, since chicken embryos were not completely ‘sensory naïve’ upon arrival. Therefore, we recommend quantifying environmental conditions during *in ovo* processing and transportation to assess how these conditions affect behavior and physiological stress responsivity in chicken embryos during late-stage incubation.

Interestingly, Broekmeulen et al. (2024) reported that FW+/L+ chicks were the only group that showed higher corticosterone levels after processing procedures (i.e., counting, grading/sorting, sexing, vaccinating), whereas chicks in other groups did not. FW+/L+ chicks were also found to have lower corticosterone levels compared to FW-/L- chicks after the treatment exposure period. These findings might indicate that both extreme photoperiod conditions (i.e., continuous light exposure (L+) vs. continuous darkness (L-)) influence corticosterone secretion through modulation of melatonin rhythmicity. Continuous light exposure and continuous darkness deviate substantially from natural incubation conditions, during which embryos are exposed to light intermittently (Zeman et al., 1999, 2004; Archer and Mench, 2014). Periodic darkness during embryonic development has been shown to be crucial for the establishment of circadian rhythms through melatonin secretion (de Jong et al., 2001; Zeman et al., 2004). Melatonin, in turn, modulates HPA activity by attenuating corticosterone surges (Saito et al., 2005), which can ultimately influence stress responsivity as well as behavioral development. Therefore, we recommend for future research to investigate effects of different light schedules during (on-farm) hatching on melatonin and corticosterone secretion, as this could improve our understanding of how varying photoperiods during incubation affect stress responsivity and behavioral development, and ultimately, overall chick welfare.

### Direction of foot and detour preferences

We also found that FW+ chicks were more likely to show a right-side foot preference compared to FW- chicks. However, it is not yet known whether a top-down or bottom-up mechanism is involved in shaping the foot preferences of chicks that received the FW+ treatment. The right-side foot preferences observed in these chicks might suggest a more positive response to testing. Specifically, a preference for the right foot could indicate that the left hemisphere was engaged during the stepping task, which typically occurs in response to familiar, non-threatening stimuli and has been associated with optimism and positive emotions (Rogers, 2010, Rogers, 2014, Rogers, 2023). Similar to the direction of foot preference, the direction of detouring was also affected by FW+ exposure. We found that FW+ chicks were more likely to detour around the barrier from the left side. In our study, we assumed that chicks detouring from the left primarily used their right eye, which indicates engagement of the left hemisphere. As with right-foot usage, right-eye usage suggests that the left hemisphere is dominant and in control of negative emotions (Rogers, 2010, 2014, 2023). Therefore, the preference for left-side detouring in FW+ chicks might again reflect a more positive response to testing and possibly the FW treatment itself.

Hollemans et al. (2018) suggested that immediate feed and water access post-hatch might have acted as an early-life enrichment, which could have influenced neural and cognitive mechanisms that are involved in emotional lateralization. The hippocampi may play a role due to their involvement in regulating the HPA axis and their responsiveness to both acute and cumulative stressors. They also exhibit pronounced structural and morphological plasticity in response to both internal and external stressors/stimuli (McKittrick et al., 2000; Keuker et al., 2003; Li et al., 2024). Moreover, environmental stimuli could add enrichment and complexity, which has been shown to stimulate hippocampal plasticity in several species (e.g., chickadees: LaDage et al., 2009; pigeons: Melleu et al., 2016), whereas chronic stressors, such as food restriction and constant light exposure, are suppressive (e.g., chickens: Robertson et al., 2017; Kumar et al., 2023; Yang et al., 2022; crows: Taufique et al., 2018). However, the specific changes in hippocampal structure and morphology that occur in response to such factors—and how these relate to visuomotor lateralization—remain unclear.

Interestingly, a study in adult captive marmosets showed that three hours of feed deprivation elevated both right hemispheric activity (as measured by tympanic membrane temperature) and blood cortisol levels, whereas after six hours of food deprivation, hemispheric activity was symmetrical again and cortisol levels were the same as in non-deprived animals (Pereira et al., 2020). These findings might support the idea that a nutritional state can influence hemispheric activity as well as stress responsivity, and consequently lateralized behavior. It might be possible that a reverse trend occurs in FW+ chicks, with an initial increase in left hemispheric activity during the early post-hatch period. However, whether this is the case, and for how long effects of early feeding might persist, remains unknown. Therefore, we recommend further investigation into the effects of early feeding on neural development, brain lateralization and cognition, with attention to when the timing of enrichment is offered

We also found that the chicks were more likely to show a right-side foot preference at seven compared to five weeks of age. However, this change in footedness between the two tests is unexpected since it has been reported that foot preferences are stable in domestic chicks after 11 days of age (Dharmaretnam et al., 2002). Possibly, foot usage was affected by repeated trials and the number of tasks in the step detour test. By design, the test involved ‘multiple dimensions’ of laterality and cognition that could be influenced differently by actual side preferences. Moreover, the chicks’ behavior could have been shaped by situational factors rather than fixed predispositions/genetics. For instance, foot side preferences could potentially be influenced by the visual assessment of the test environment, as observed for handedness in humans (Ocklenburg et al., 2010).

### Strength of detour preferences

Surprisingly, the total number of chicks with a detour preference changed with age (from 108 to 124 chicks). Additionally, the strength of detour preferences increased with age, which was also found in a previous study by Broekmeulen et al. (2025). It is unclear what mechanism might have caused changes in detour preferences between the two tests. However, the visual assessment of the test environment and social stimuli might have affected the detour preferences (Vallortigara et al., 1999). For instance, in quail, another *Galliformes* species, detour responses were influenced by familiarity of the social stimulus and the number of trials (Zucca and Sovrano, 2008). The detour preferences in the chicks might not reflect true lateralized response but a strong motivation for social reinstatement (Vallortigara et al., 1990; Regolin et al., 1994), as well as learning contingencies during previous trials, which could alter their detour choices in subsequent test trials (van Horik et al., 2018; Rosenberger et al., 2021). This reasoning is supported by the short latencies to leave the start box and short duration to finish (see below).

### Latency to leave the start box and duration to finish the test

Observations on latency to leave the start box and duration to finish the test were included in the step detour test to assess fearful behavior in focal chicks and to compare latencies between chicks from different groups. A previous study that focused on effects of related hatching systems factors (Broekmeulen et al., 2024) did not find treatment differences during the tests for latency to leave the start box or duration to finish the step detour test. Therefore, it was decided to include observations on latency to leave the start box on the first day of habituation when no social stimulus was present. Based on literature (e.g., Sui et al., 1997; Andrew et al., 2009; Rogers, 2012; Archer and Mench, 2017), it was anticipated that dark-incubated chicks (i.e. FW-/L- and FW+/L-), would be more fearful and thus take longer to leave the start box and finish the test compared to light-incubated chicks (i.e., FW+/L+ and FW-/L+). Against expectations, we did not find L treatment factor differences for latency to leave the start box on the first day of habituation. The novelty of being alone in the start box might have resulted in an absence of treatment factor effects. In addition, we observed that the chicks got quicker to leave the start box and to finish the test with each day of testing, as previously found by Broekmeulen et al. (2025), likely as the chicks learned where the conspecifics were and they were highly motivated to join them (e.g., social stimulus) (Suarez and Gallup, 1983; Väisänen and Jensen, 2003). However, there is a knowledge gap regarding the linkage between motivation and behavioral lateralization in non-human animals.

## Conclusion

In conclusion, light-incubated chicks, independent of immediate access to feed and water after hatch, were faster to detect the predator and to return to pecking compared to dark-incubated chicks. These findings suggest that light-incubated chicks, in comparison to dark-incubated chicks, have an increased ability to control negative behavioral responses and are quicker to assess if a novel stimulus in the environment could pose a threat.

In a commercial setting, improved multitasking abilities could aid chicks to better adapt to stressful aspects of their daily environment and thereby improve their welfare during the rearing period. Based on the multitasking test, we could not confirm interaction or modulating effects of immediate post-hatch feed and water access on the behavioral and cognitive development.

The step detour test revealed effects of light during late incubation (L+) on strength of foot preferences. Immediate feed and water access (FW+) and week of age were found to influence the direction of footedness as well as the direction of detouring. The right-side preferences during testing suggest a more positive response to testing, which typically occurs in response to familiar, non-threatening stimuli and has been associated with optimism and positive emotions.

The treatment factor differences in foot and detour preferences could potentially be explained by short- and long-term modulation of brain lateralization by interactions between top-down and bottom-up mechanisms that underly light-induced lateralization and emotional lateralization. How visuomotor lateralization as well as emotional lateralization is modulated by different environmental enrichments or stimuli and how these interact merits further investigations.

## Disclosures

The authors declare that they have no known competing financial interests or personal relationships that could have appeared to influence the work reported in this paper.

## CRediT authorship contribution statement

**Catharina M.H. Broekmeulen:** Writing – review & editing, Writing – original draft, Visualization, Methodology, Investigation, Data curation, Conceptualization, Formal analysis. **Sabine G. Gebhardt-Henrich:** Writing – review & editing, Supervision, Methodology, Conceptualization. **Yamenah Gómez:** Writing – review & editing, Visualization, Methodology, Formal analysis, Data curation. **Michael J. Toscano:** Writing – review & editing, Validation, Supervision, Resources, Project administration, Funding acquisition, Conceptualization, Methodology.
